# A Dynamic Framework for Internet-Based Network Time Protocol

**DOI:** 10.3390/s24020691

**Published:** 2024-01-22

**Authors:** Kelum A. A. Gamage, Asher Sajid, Omar S. Sonbul, Muhammad Rashid, Amar Y. Jaffar

**Affiliations:** 1 James Watt School of Engineering, University of Glasgow, Glasgow G12 8QQ, UK; 2 Deanship of Scientific Research, Umm Al Qura University, Makkah 21955, Saudi Arabia; malikasher267@gmail.com; 3 Computer Engineering Department, Umm Al Qura University, Makkah 21955, Saudi Arabia; ossonbul@uqu.edu.sa (O.S.S.); ayjaafar@uwu.edu.sa (A.Y.J.)

**Keywords:** network time protocol, time synchronization, GPS-based network time protocol, internet-based network time protocol, FPGA

## Abstract

Time synchronization is vital for accurate data collection and processing in sensor networks. Sensors in these networks often operate under fluctuating conditions. However, an accurate timekeeping mechanism is critical even in varying network conditions. Consequently, a synchronization method is required in sensor networks to ensure reliable timekeeping for correlating data accurately across the network. In this research, we present a novel dynamic NTP (Network Time Protocol) algorithm that significantly enhances the precision and reliability of the generalized NTP protocol. It incorporates a dynamic mechanism to determine the Round-Trip Time (RTT), which allows accurate timekeeping even in varying network conditions. The proposed approach has been implemented on an FPGA and a comprehensive performance analysis has been made, comparing three distinct NTP methods: dynamic NTP (DNTP), static NTP (SNTP), and GPS-based NTP (GNTP). As a result, key performance metrics such as variance, standard deviation, mean, and median accuracy have been evaluated. Our findings demonstrate that DNTP is markedly superior in dynamic network scenarios, a common characteristic in sensor networks. This adaptability is important for sensors installed in time-critical networks, such as real-time industrial IoTs, where precise and reliable time synchronization is necessary.

## 1. Introduction

Time synchronization is a key requirement in real-time industrial IoT-based systems and other time-critical applications, such as cyber-physical systems [1], power grids [2], financial systems [3], transportation systems [4], and smart cities security applications [5]. Even a slight deviation in time reference can induce significant faults in these systems, and therefore, precise and reliable time synchronization is crucial [6,7]. One of the most widely used protocols for time synchronization in computer networks is the Network Time Protocol (NTP) [8]. It works by exchanging data packets between clients/servers and selects the best time source from a set of potential sources followed by the local clock adjustments to match the selected source.

There are two basic types of NTP, namely Internet-based NTP (INTP) and GPS-based NTP (GNTP). The INTP relies on a network of NTP servers connected to the Internet [9]. It is accepted as a reliable, scalable, and widely used protocol for time synchronization. A global network of NTP servers (“pool.ntp.org”), distributed across various locations worldwide, is commonly used to obtain accurate time synchronization. Therefore, any NTP client can obtain time information from the most appropriate and reliable servers by connecting to multiple geographically distributed servers from the pool, at any specific time. This approach enhances the overall accuracy and resilience of time synchronization [10]. Nevertheless, internet-based NTP has several issues, including network congestion, packet delay, and clock drift. Furthermore, it is susceptible to exploitation, including Distributed Denial of Service (DDoS) attacks, which have the potential to degrade service and endanger the precision of timing signals [11].

On the other hand, GNTP approach precisely synchronizes clocks for the network timing reference. It employs signals from the Global Positioning System (GPS) [12]. Atomic-clock-equipped GPS satellites send extremely precise time information with a range uncertainty of about 5 ns. Upon receiving these signals, network devices can accurately detect their time information and adjust their internal clocks accordingly. As a result, all of the network’s devices are ensured to have a precise and constant time reference with a master clock.

### 1.1. Research Gap

As stated previously, the issues with the INTP approach are network congestion, packet delay, and clock drift. These issues (variations) result in a varying round trip time (RTT), and therefore, pose a challenge to time synchronization. The RTT is the time taken by a network packet to travel from the client to a server and back. Particularly, varying RTT causes timekeeping inconsistencies between different network devices. Consequently, it compromises the accuracy of synchronization. Therefore, there is a need for adaptive (dynamic) protocols that can manage dynamic RTT to enhance synchronization. As a result, an increasing amount of research is being conducted to improve the security, accuracy, and precision of GNTP as well as INTP synchronization protocols. The details of the related research for GNTP as well as INTP will be provided in Section 2 of this article. Nevertheless, there is a lack of comprehensive comparison between GNTP and INTP in terms of various performance parameters, such as accuracy, dependability, and security.

### 1.2. Proposed Solution and Achieved Results

To address the limitations of the traditional static NTP (SNTP), we have proposed a DNTP protocol by considering the continuous monitoring and adaptation of real-time variations in network delay. The major steps of the proposed DNTP protocol are: (1) the acquisition of real-time RTT measurements, (2) the analysis of dynamic fluctuations in the network delay (jitter), and (3) the incorporation of these real-time RTT measurements into the time synchronization process. In other words, by using real-time RTT values, the proposed DNTP protocol adjusts its synchronization algorithm to account for changing network conditions. Therefore, it results in improved accuracy and reliability of time synchronization over the network.

For the validation of our approach, a comparison between SNTP, DNTP, and GNTP has been conducted. The comparison is based on the statistical analysis of the mean, variance, median, and standard deviation of each protocol. These parameters are used as a quantitative measure to evaluate the accuracy and performance of the time synchronization achieved by each protocol. For data collection and statistical analysis, Python and MATLAB have been used, respectively. Similarly, synchronization signals have been taken from the devices implementing the respective network protocols. Subsequently, performance metrics (such as standard deviation, variance, mean, and median) have been computed for comparative analysis. The combination of Python and MATLAB platforms, along with the selected metrics, has enabled us to validate and compare the performance of SNTP, DNTP, and GNTP approaches efficiently.

### 1.3. Outcomes and Significance

The proposed DNTP has demonstrated lower variance ( $2.370\times 10^{8}$ μ $s^{2}$ ) and standard deviation ( $1.540\times 10^{4}$ μ s) when compared to SNTP (variance: $7.422\times 10^{8}$ μ $s^{2}$ , standard deviation: $2.724\times 10^{4}$ μ s). Although SNTP demonstrates a marginally higher mean accuracy ( $8.899\times 10^{3}$ μ s), its negative median value ( $-8.380\times 10^{3}$ μ s) raises concerns regarding the precise synchronization in dynamic network scenarios. The GNTP results indicate a variance of $7.663\times 10^{6}$ μ $s^{2}$ , a standard deviation of $2.768\times 10^{3}$ μ s, a mean of $5.142\times 10^{4}$ μ s, and a median of $1.8251\times 10^{4}$ μ s.

Rigorous statistical analyses in this article provide valuable guidance for selecting the optimal NTP method, advancing time synchronization in sophisticated computer networks. From the achieved results, it can be concluded that the DNTP is preferable in all those applications where precise synchronization is critical with varying network conditions. On the other hand, the precision is better in SNTP but it is only suitable for less dynamic environments. To summarize, the proposed DNTP approach improves precision and reliability by addressing the variable RTT limitation.

### 1.4. Organization

The present work is organized as follows. The theoretical background on NTP is provided in Section 2. Section 3 introduces the novel dynamic RTT-based NTP framework, addressing challenges in time synchronization. Section 4 presents key findings of our proposed approach, demonstrating practical effectiveness. Finally, the article is concluded in Section 5, which highlights the significance of our contributions and potential implications.

## 2. Background and Related Work

The mathematical formulations for both INTP and GNTP are presented in Section 2.1. The architectures for INTP and GNTP are presented in Section 2.2 and Section 2.3, respectively. The related work for both INTP and GNTP is discussed in Section 2.4 and Section 2.5, respectively. Finally, the novelty of our work is presented in Section 2.6.

### 2.1. Mathematical Preliminaries

**Figure 1:**
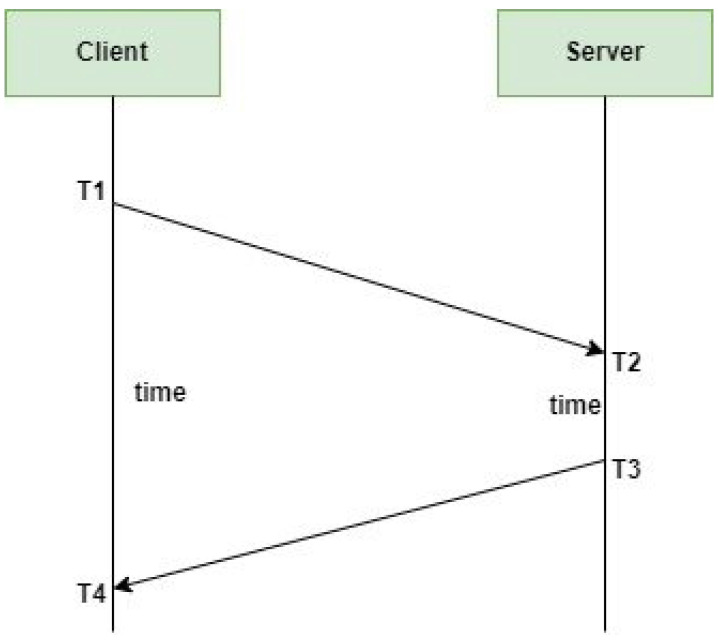
NTP packet exchange between client and server.

Networked devices use NTP to synchronize their clocks in internet-based and GPS-based systems. Synchronization is accomplished through the exchange of data packets between a client and a server. The server receives a request packet from the client. It replies with a packet that includes its own time. It is dependent on a very accurate source of time in the form of a reference clock. The reference clock can be an atomic clock, radio clock, or GPS receiver. The synchronization process is shown in Figure 1 and uses the following formula to calculate the offset ΔT :

(1) $$\Delta T=\frac{\left(T2-T1)+(T3-T4\right)}{2}$$

where T1 indicates the client timestamp when the request packet is sent, T2 corresponds to the server timestamp when the request packet is received, T3 denotes the server timestamp when the reply packet is sent, and T4 signifies the client timestamp when the reply packet is received.

Once the offset ΔT is calculated, the time spent with the client is estimated using the equation below:

(2) $$T_{est}=T2+\Delta T$$

It is important to remember that when packets are transferred between the server and client, the mathematical formulae utilized in both GPS-based and internet-based NTP protocols include the same underlying principle. Therefore, Equations (1) and (2) can be used for both cases.

### 2.2. Basic INTP Architecture

**Figure 2:**
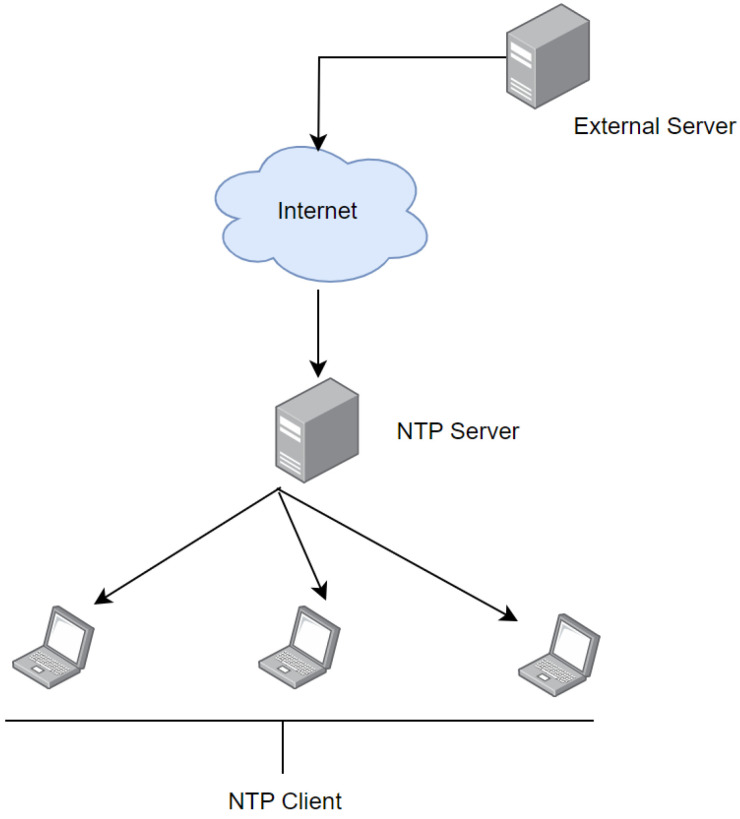
Working mechanism of a typical INTP architecture.

The basic internet-based NTP architecture is shown in Figure 2. It involves time synchronization across a network using an external NTP server and a local NTP server. The time is obtained from an external server available over the Internet (pool.ntp.org). Then, a local NTP server is set up within the network. This local server acts as a middleman to facilitate time synchronization. It periodically retrieves time from the external server and distributes it to the client network. These client networks, known as NTP clients, synchronize their clocks with the local NTP server. It ensures consistent and precise time across the network [13].

### 2.3. Basic GNTP Architecture

**Figure 3:**
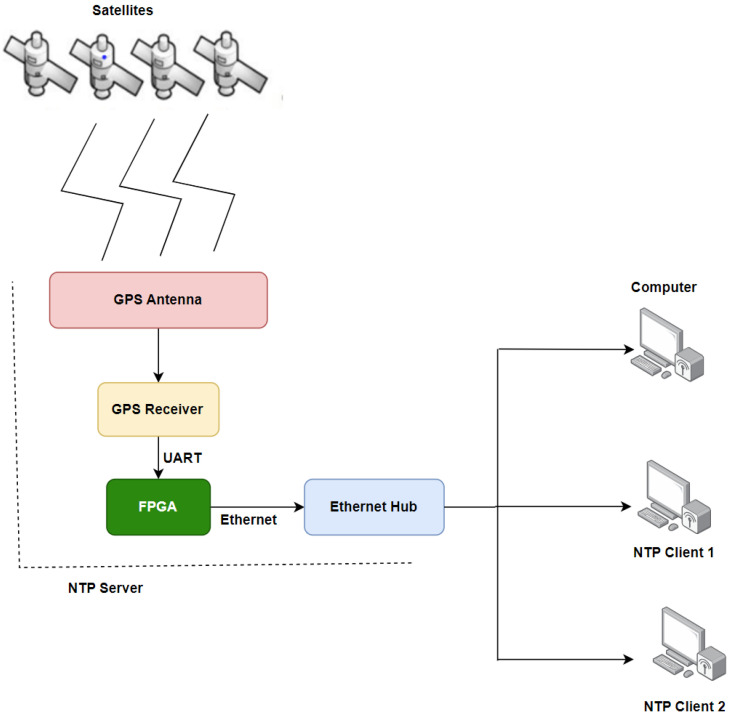
Working mechanism of a typical GNTP architecture.

The architecture of the system is illustrated in Figure 3. It can be noticed that the time data originate from the GPS satellites. Subsequently, it is received by a GPS receiver with a CSAC (Chip-Scale Atomic Clock) via a GPS antenna. The received time data are further processed by an FPGA (Field Programmable Gate Array). A universal asynchronous receiver–transmitter (UART) module is used for this purpose. If a client node requests the time data, the FPGA will transmit the processed time data via Ethernet.

### 2.4. Existing Architectures for Internet-Based NTP (INTP)

In the last few decades, the INTP has been frequently employed for time synchronization in computer networks. Therefore, multiple studies have been conducted to enhance its accuracy and security for multiple applications. For example, the authors in [14] have proposed an INTP detection module for DDoS attacks. With the use of continuous NTP traffic monitoring, this module spots unusual request rates coming from various sources (a feature that is frequently present in DDoS attacks). As a result, it effectively improves IoT security by detecting and preventing DDoS attacks. While proactive threat mitigation, smooth integration with current NTP systems, and real-time detection are the benefits of this strategy, disadvantages may include false positives and evasion by sophisticated attacks.

The possible hazards related to unauthenticated NTP communication have been studied in [15]. It investigates a variety of attack scenarios, including both on-path and off-path assaults, such as time manipulation using IPv4 packet fragmentation and DDoS operations. Moreover, simple countermeasures to improve NTP security have also been suggested. The work in [15] is significant in the sense that it points out a few possible NTP vulnerabilities and provides advice on how to mitigate them. However, to evaluate the effects of these attacks in practical settings and create more all-encompassing defense plans, additional research is required.

Similarly, the authors in [16] addressed the difficulty of preserving perfect communication and cooperative processing between sensors through time synchronization in the field of Wireless Sensor and sensor/actuator networks. To analyze and assess the performance and efficiency of several synchronization algorithms for energy-efficient information processing and routing in wireless sensor networks (WSNs), the research examines both quantitative and qualitative factors. This research offers a detailed analysis of different synchronization protocols used in WSNs, outlining their strengths and weaknesses. Such information is crucial for decision makers to identify the most suitable protocols for specific WSN applications. However, there are challenges, such as the need for detailed data analysis and empirical assessments, especially considering the scalability of these protocols for larger WSN systems and their ability to adapt to dynamic network conditions [17]. Overall, this research contributes to improving time synchronization in computer networks, enhancing security, and increasing efficiency across various network types, such as IoT and WSNs.

### 2.5. Existing Architectures for GPS-Based NTP (GNTP)

GPS time synchronization has been widely used as an accurate timing reference in computer networks. A GPS time-sync method for seismic surveys with high precision is introduced in [18]. By installing GPS receivers at survey locations to synchronize seismic data capture equipment with nanosecond-level precision, this technology offers advantages such as precise data gathering and energy efficiency. Another low-power GPS synchronization method for wireless nodes is presented by [19]. Similar to the work in [18], this technique also ensures precise and continuous coordination using GPS signals to synchronize the clocks in wireless nodes. It ultimately enhances the network performance and reduces errors. However, the limitation of the works in [18,19] is their inability to receive signals in those locations where GPS is prohibited.

The application of GPS technology for navigation and location has also been studied in [20]. It utilizes GPS receivers to accurately determine the location and speed of the device, facilitating precise navigation and route planning. Similarly, the work in [21] introduces a time synchronization method for transmission substations, integrating GPS with IEEE 1588 (Precision Time Protocol). While the methods in [20,21] achieve high precision in time synchronization by employing GPS receivers located at substations, the method’s dependence on external GPS signals can pose limitations in certain environments. They might face challenges in areas with weak GPS signal reception, despite their high precision and extensive coverage.

In short, existing architectures for GNTP [18,19,20,21] highlight the significant role of GPS technology in network coordination, navigation, and time synchronization. While these innovations enhance the efficiency, accuracy, and reliability of various systems and applications, their reliance on external GPS infrastructure and signals means that their suitability must be carefully evaluated in specific application scenarios.

### 2.6. Novelty

This article proposes an innovative approach to enhance the accuracy and reliability of INTP by incorporating dynamic RTT. Through active monitoring and adaptation to changing network delays, the proposed approach improves synchronization stability and addresses the limitations of static measurements, contributing to the development of more robust and adaptive time synchronization protocols.

## 3. Proposed Methodology

This section presents a dynamic mechanism of internet-based NTP (DNTP) for time synchronization. In Section 3.1, we present the motivation for DNTP. Section 3.2 and Section 3.3 provide structural and behavioral representations of the proposed DNTP framework. Finally, in Section 3.4, the advantages and applications of the DNTP are discussed.

### 3.1. Motivation for DNTP

As mentioned previously in Section 1.1, RTT is the time taken by a network packet to travel from a client to a server and back. In other words, it indicates the network delay and total latency. Therefore, the time difference ( Δ ) between the server and the client in SNTP is explained by Equation (3):

(3) $$\Delta =\frac{\left(T2-T1)+(T3-T4\right)}{2}$$

where the timestamps T1 and T2 are used for client-server timestamped transfers and the timestamps T3 and T4 are used for server-side timestamped transfers. In addition to these four timestamped variables, there must be a set threshold value *T* for the time synchronization to be successful. This implies that a successful time synchronization is achieved if the time offset is smaller than the predetermined threshold (*T*). However, the static threshold may lead to incorrect time synchronization as it is not suitable to adapt to changing and variable network conditions.

### 3.2. Structural Representation of DNTP Framework

**Figure 4:**
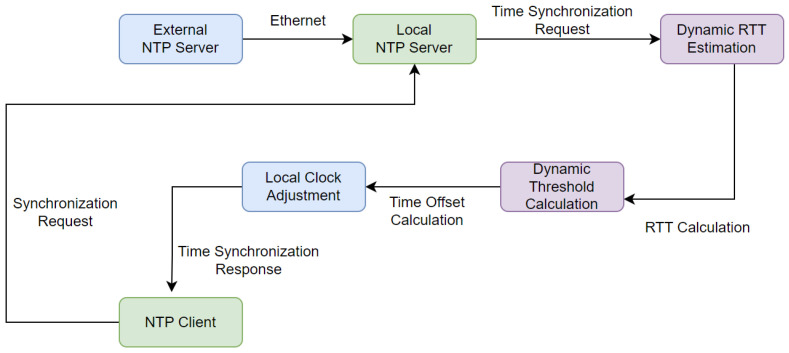
DNTP framework using RTT-based time synchronization.

The dynamic RTT-based time synchronization process is illustrated in Figure 4. The process starts with the external NTP server which acts as the primary time reference. The external NTP Server receives accurate time information from a pool of NTP servers (“pool.ntp.org”). It provides highly precise time signals from diverse and reliable sources.

The local NTP server is implemented on an FPGA. It is connected to the external NTP server via Ethernet. Initially, an NTP client sends a synchronization request. In response to this request, the dynamic RTT estimation module calculates the RTT. The RTT is calculated by measuring the time taken for the request to reach the external NTP server as well as the time taken by the response to return.

The calculated RTT is then used for precise time synchronization. The dynamic threshold calculation module determines the dynamic threshold (*D*) by computing the standard deviation of recent RTT samples. This dynamic threshold induces adaptability to change according to network conditions. With the RTT and dynamic threshold together, the local clock adjustment calculates the time shift required for the synchronization, which is thereby used to update the client’s local clocks accordingly.

The NTP client actively participates in the time synchronization process by updating its own time based on the received response from the local NTP server. After updating its local clock, the NTP client sends another time synchronization request to the local NTP server. The request includes the client’s new local time information. Subsequently, the NTP client waits for a response containing the accurate time information from the external NTP server. This iterative process ensures continuous and precise time synchronization within the network.

### 3.3. Behavioral Representation of the DNTP Framework

In the proposed DNTP framework, the time difference ( Δ ) between the client and server clocks is determined using the following method:

(4) $$\Delta =\left(\frac{\left(T2-T1)+(T3-T4\right)}{2}\right)-D$$

where D=k×σ , where *D* represents the dynamic threshold, *k* is a constant factor explained in Algorithm 1, and σ represents the standard deviation of the observed RTT values. The dynamic threshold, represented by *D*, reflects the variability in RTT values and allows for adaptability in the time synchronization process. By considering the RTT variability, Algorithm 2 can dynamically adjust the synchronization mechanism to accommodate changing network conditions and jitter in communication delays. To ensure reliable time synchronization, we also determine an appropriate fixed threshold value (*T*) for dynamic RTT. This fixed threshold serves as a reference for successful synchronization, even in dynamic network scenarios. The calculation of the fixed threshold is as follows:

1. The time synchronization precision level, often expressed in milliseconds or microseconds, is defined as the desired accuracy.
2. Variations in network circumstances can cause variations in RTT values. A safety margin is employed to ensure strong synchronization by preventing unexpected variations.
3. The safety margin is subtracted from the desired accuracy to determine the fixed threshold, as shown in Equation (5):

(5) T=DesiredAccuracy−SafetyMargin

#### 3.3.1. Algorithm for Determining the Constant Factor *k* in DNTP Framework

Algorithm 1 computes the constant factor *k* using three inputs: (1) a set of recorded round trip time (RTT) values that provide an overview of the network’s latency, (2) a safety margin that serves as a buffer, and (3) the desired accuracy level, expressed in milliseconds or microseconds. A safety margin is essential to ensure synchronization stability, whereas an accuracy level sets the synchronization precision goal.
**Algorithm 1:** Determining the Constant Factor *k*
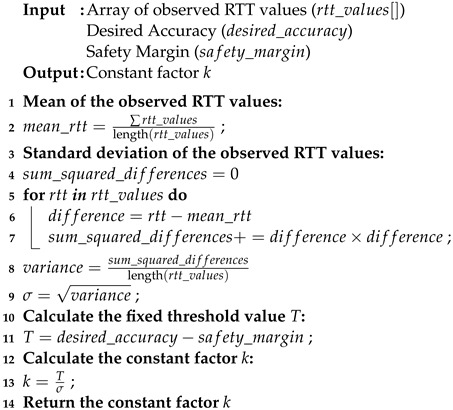


First, the average round trip time ( mean_rtt ) is computed by adding up all the observed RTT values and dividing by the total number of observations. Secondly, the standard deviation ( σ ) is computed, which reveals how much the RTT values deviate or spread from the mean. Subsequently, the fixed threshold *T* is obtained by subtracting the safety margin from the target accuracy to preserve a reliable synchronization even in fluctuating network conditions. The last step is to determine the constant factor *k* (dividing *T* by the standard deviation).

#### 3.3.2. Algorithm for RTT Estimation in DNTP Framework

Algorithm 2 aims to improve time synchronization accuracy. It operates continuously, measuring the RTT between the NTP client and the server. The process begins by initializing an array for storing RTT samples and setting a threshold value (*T*). It also establishes an initial dynamic threshold value (*D*). During each cycle of the loop, the NTP client records the current time ( time_sent ) before sending a request to the server. Once the server responds, the client again logs the time ( time_received ). The RTT is calculated as the difference between time_received and time_sent . This RTT value is then stored in the array for use in later calculations.
**Algorithm 2:** Dynamic RTT Estimation in NTP**Input**: NTP client and server connected over the network; NTP packet exchange mechanism **Output**: Real-time RTT estimation (RTT) 
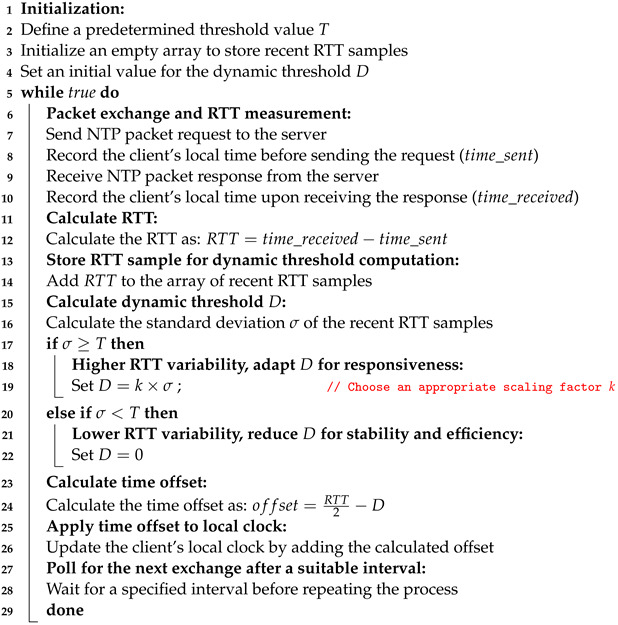


The dynamic threshold (*D*) is computed according to two different scenarios: (1) If the standard deviation ( σ ) is greater than the threshold value (*T*), showing an increased RTT variability, a suitable scaling factor *k* is applied to the dynamic threshold ( D=k×σ ). The application of the scaling factor offers responsiveness to changing network conditions. (2) If the standard deviation of the RTT is low or equal to the threshold, showing some stable RTT values, the algorithm sets the dynamic threshold to zero D=0 . The algorithm then computes the time offset (network delays) as $offset=\frac{RTT}{2}-D$ . This computed offset is used to adjust the client’s clock to match the server’s time more accurately. The algorithm repeats itself after a specified interval of time to maintain accurate synchronization. Therefore, with a dynamic estimation of the RTT and an adaptive adjustment of the time offset, it enhances the precision and reliability of time synchronization.

### 3.4. Pros and Cons of DNTP for Time Synchronization

The DNTP framework offers several advantages in terms of accuracy and responsiveness. By dynamically adjusting the time offset based on the RTT threshold, it ensures the agility of the synchronization process in response to changing network conditions. Consequently, it maintains a precise track of network timing. This adaptive behaviour allows the optimal utilization of system resources and time synchronization in both normal and highly variable network environments. At the same time, DNTP suffers from some limitations as the continuous monitoring and adaptation of the offset calculation may add further computational overhead, which can potentially impact resource utilization and power budget, especially when a large number of network devices are connected. Despite some limitations, the advantage of improved accuracy and adaptive behaviour results in precise time reference for applications where time synchronization is required. This is particularly true for distributed systems and telecommunication networks, which get considerable benefit from the enhanced reliability offered by our dynamic RTT-based NTP method. By carefully weighing the trade-offs between computational overhead and synchronization performance, our approach presents a robust solution for achieving accurate time synchronization in various real-world applications.

## 4. Results and Discussion

This section presents performance analysis results for the proposed method (DNTP) and compares them with SNTP and GNTP methods. The utilized performance metrics are variance, standard deviation, mean, and median. The experimental setup is explained in Section 4.1. Similarly, the data collection and analysis procedures are provided in Section 4.2. Subsequently, a comprehensive performance evaluation is provided in Section 4.3. Section 4.4 and Section 4.5 present the graphical analysis and tabular comparison for various approaches (SNTP, DNTP, and GNTP), respectively. Finally, some probable confusions (pitfalls) in mean accuracy results and variability in time synchronization have been briefly discussed in Section 4.6.

### 4.1. Experimental Setup

**Figure 5:**
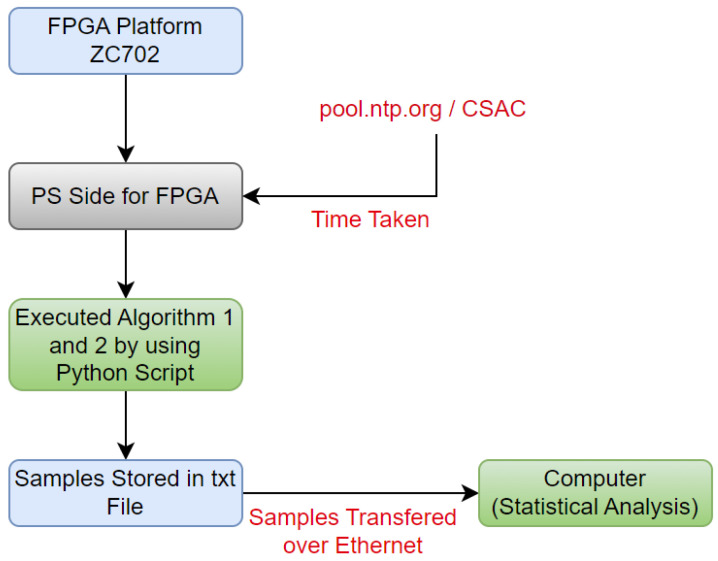
Experimental setup.

Figure 5 shows a comprehensive time synchronization procedure using the ZC702 FPGA platform. The Processing System (PS) on the FPGA is employed to facilitate the synchronization from both NTP sources (internet-based as well as GPS-based). The PS side of FPGA engages with the “pool.ntp.org” server for internet-based NTP synchronization. Highly accurate time data are provided by the CSAC, which is utilized for GPS-based synchronization. To improve the overall synchronization accuracy, the CSAC and FPGA integration is essential for obtaining exact time signals from GPS sources. The time taken by these sources is recorded for subsequent analysis.

The execution of Algorithms 1 and 2 is carried out through Python scripts. This allows an effective interfacing of the FPGA platform with external sources. The obtained synchronization samples are stored in a text file, ensuring data integrity. These samples are then transmitted over Ethernet, establishing a seamless connection to a computer for comprehensive analysis. On the computer, a robust statistical analysis is performed using MATLAB. This analytical phase yields insights into the synchronization accuracy, in terms of variance, standard deviation, mean, and median.

### 4.2. Data Collection and Analysis

For data collection on both the internet-based and GPS-based NTP synchronization methods, we used Python, processing 10,000 samples for each technique. Python was chosen for its user-friendliness and adaptability in managing data. The statistical analysis has been carried out using MATLAB, a popular tool for data analysis. Its ability to quickly process large datasets and its various analytical tools are crucial for identifying key trends and insights from our data. The use of Python and MATLAB is instrumental in analyzing the performance data from these synchronization techniques. Their combined efficiency and reliability has provided the necessary tools to draw meaningful insights and conclusions. Finally, the hardware platform for data collection utilizes FPGA technology to guarantee precise and accurate data handling. This approach enabled us to effectively evaluate the accuracy and consistency of synchronization in both GPS-based and internet-based NTP.

### 4.3. Performance Metrics

Three NTP methods have been compared for accuracy and time synchronization in Table 1. Variance, standard deviation, mean accuracy, and median accuracy are the metrics used in the evaluation process. Mean accuracy denotes the average synchronization accuracy, whereas variance and standard deviation show the variability and spread of synchronization findings. The central tendency of the synchronization outcomes is shown in the median accuracy. These metrics provide valuable insights into the performance of each NTP approach, aiding in the selection of the most suitable method for specific applications and network conditions.

### 4.4. Graphical Analysis

**Figure 6:**
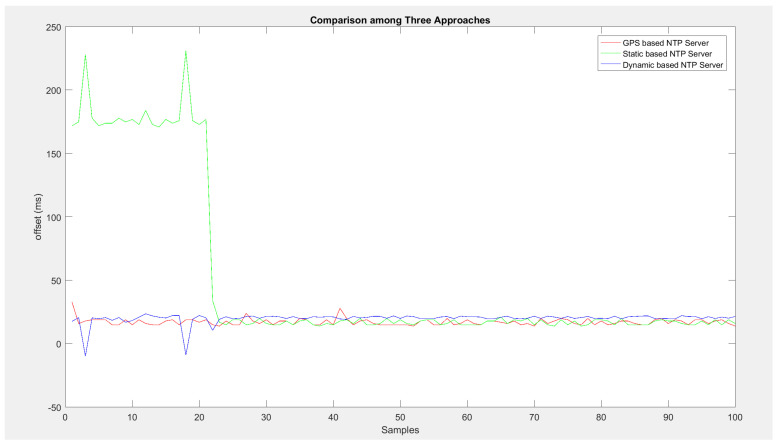
Comparisons of GPS-based, static, and dynamic NTP algorithms.

In Figure 6, we provide a comprehensive analysis of three distinct strategies employed for NTP synchronization: namely, GPS-based, static-based, and dynamic-based NTP servers. The graph’s x-axis represents the sequence of collected samples, while the y-axis signifies the time offset measured in milliseconds. To ensure clarity, the graph focuses on a subset of 100 samples from a larger dataset of 10,000. Upon closer inspection, discernible patterns emerge, illustrating the distinctive behaviors of each synchronization approach. The static-based NTP server’s trajectory, depicted in green, initially exhibits notable spikes. These spikes correspond to instances where the network experiences temporary disruptions, leading to substantial deviations in time measurements. Over time, these spikes diminish in magnitude, but there is still a wavy pattern. This shows that the time reporting is not steady, especially when the network changes.

On the other hand, the red trajectory shows the GNTP approach. It is clear from the graph that a consistent trajectory follows right from the beginning. There are some minor oscillations, but they are short-lived. Finally, the graph in blue color represents the DNTP approach. This approach employs adaptable threshold calculations to determine time offsets. It results in smaller deviations as compared to the SNTP approach. Consequently, the DNTP trajectory is smoother with fewer pronounced spikes.

Overall, the DNTP approach emerges as the most effective among the three methodologies. Its ability to dynamically adjust threshold values leads to lower time offsets. As a result, resilience to network-induced disruptions increases. It is important to note that the GNTP approach shows very good stability; however, it is the DNTP approach that achieves an optimal balance between stability and adaptability, making it particularly well-suited for environments characterized by fluctuating network conditions.

### 4.5. Tabular Analysis

This section is further subdivided into three subsections. Section 4.5.1 provides a comparison between SNTP and DNTP approaches. Section 4.5.2 provides a comparison between SNTP and GNTP approaches. Section 4.5.3 provides a comparison between GNTP and DNTP approaches.

#### 4.5.1. Comparison between SNTP and DNTP

The comparison between the SNTP and the DNTP methods reflects a significant distinction in the time synchronization performance. The DNTP approach demonstrates a remarkable advantage over SNTP in terms of consistency and precision. This fact can be observed from the lower variance ( $2.370\times 10^{8}$ μ $s^{2}$ ) and standard deviation ( $1.540\times 10^{4}$ μ s) of the DNTP approach as compared to the SNTP, which has a relatively higher variance ( $7.422\times 10^{8}$ μ $s^{2}$ ) and standard deviation ( $2.724\times 10^{4}$ μ s). It allows DNTP to successfully represent stable and precise timekeeping across varying network conditions. Moreover, the mean accuracy of the DNTP is $6.0642\times 10^{4}$ μ s, while the mean accuracy for the SNTP is $8.899\times 10^{3}$ μ s. It implies that the values of mean accuracy are higher for the DNTP as compared to the SNTP. This apparent contradiction can be reconciled by considering the operational environments of the two systems. The DNTP is designed to function in more variable and challenging network conditions where maintaining synchronization is inherently more difficult. Therefore, a slightly higher mean accuracy in such conditions does not necessarily indicate poorer performance.

It is also important to highlight an interesting finding about the median accuracy. The SNTP approach shows a negative median accuracy of $-8.380\times 10^{3}$ microseconds, while the DNTP offers a median accuracy of 66,956 microseconds, showing reliable synchronization for most applications. The negative value suggests that the SNTP approach may show significant time-synchronized differences, particularly in situations involving dynamic networks. The SNTP has limits, particularly when dealing with dynamic RTT and changing network conditions, as shown by this inconsistent median accuracy. Therefore, the DNTP approach is a more appropriate and dependable option due to its stable performance and adaptability for applications that require consistent and dependable time synchronization. To summarize, compared to the SNTP approach, which has limits in dynamic network situations, the DNTP approach has greater consistency, precision, and mean accuracy.

#### 4.5.2. Comparison between SNTP and GNTP

The comparison between the SNTP and the GNTP methods exhibits distinct performance characteristics for the accuracy of time synchronization. The GNTP shows a considerable lower variance ( $7.663\times 10^{6}$ μ $s^{2}$ ) and standard deviation ( $2.768\times 10^{3}$ μ s) as compared to the SNTP variance ( $7.422\times 10^{8}$ μ $s^{2}$ ) and standard deviation ( $2.724\times 10^{4}$ μ s). This means that the GNTP provides greater consistency and precision in time synchronization, because of its lower variability in accuracy. Similar to the DNTP approach, the values of mean accuracy are higher for the GNTP as compared to the SNTP. Again, this apparent contradiction can be reconciled by considering the operational environments of the two systems. Another parameter, i.e., the median accuracy of the GNTP ( $1.8251\times 10^{4}$ μ s), is also higher than that of the SNTP ( $-8.380\times 10^{3}$ μ s), which further confirms the superior performance of the GNTP in maintaining precise synchronization.

It is essential to consider that the GNTP shows a slightly lower variance and standard deviation than the DNTP, which suggests that the GNTP has a minor tendency of variability in synchronization accuracy, although still lower than the SNTP. The better performance of GNTP can be attributed to the inherent characteristic of Global Positioning System (GPS) signals, which provide highly accurate time reference. In general, the above comparison highlights the advantages of GNTP over SNTP in terms of consistency, precision, and mean accuracy. Utilizing GPS signals for time referencing enables GNTP to achieve higher stability in time synchronization of network entities.

#### 4.5.3. Comparison between DNTP and GNTP

The GNTP exhibits superior precision and consistency, with lower variance and standard deviation values. Its mean accuracy is also slightly lower than the DNTP approach. The reason is that the GNTP relies on highly accurate time information from GPS satellites. On the other hand, leveraging a dynamic threshold based on standard deviation, the DNTP approach adapts to changing network conditions. The comparison highlights the distinct advantages of both DNTP and GNTP in different scenarios. The DNTP stands out for its dynamic threshold mechanism, enabling it to adeptly adapt to real-time network changes. This feature makes the DNTP particularly effective in environments with high network variability, where its ability to consistently adapt is essential. On the other hand, the GNTP is the preferred option in scenarios requiring reliable external time references. Its use of precise GPS satellite data ensures consistent and stable time synchronization. By understanding these specific strengths, decision makers can better choose the most suitable NTP approach, enhancing time synchronization performance in complex computer networks according to the unique needs and constraints of each network.

### 4.6. Discussion on Mean Accuracy and Variability in Time Synchronization

In this subsection, we address a probable source of confusion (pitfall) by discussing the mean accuracy of GNTP, DNTP, and SNTP approaches. A higher mean accuracy can be observed in the GNTP approach as compared to the SNTP approach, which at first glance may indicate less precision. This higher value, however, actually reflects the operational environment of GNTP, which is characterized by recurrent satellite signal delays. In other words, it is not an indication of poor performance; rather, it highlights specific challenges associated with satellite-based synchronization. Similarly, the DNTP approach, which is designed for dynamic and varying network conditions, demonstrates higher mean accuracy in comparison to the SNTP approach. This rise demonstrates DNTP’s adaptability to network modifications, an essential quality in dynamic environments. The higher mean accuracy of GNTP and DNTP in their respective environments indicates their applicability and effectiveness.

In addition to the mean accuracy, the DNTP approach exhibits greater variation and standard deviation when compared to the GNTP approach. In this case, high DNTP metrics should not be interpreted as evidence of inaccuracy. Rather, they demonstrate DNTP’s ability to adjust and react to changing and dynamic network conditions. One of DNTP’s main advantages is its versatility, which makes it appropriate for situations where the network circumstances are continually changing. Therefore, we have emphasized throughout our work that the operational conditions of these protocols must be considered when evaluating them.

## 5. Conclusions

This article has proposed an FPGA implementation of a DNTP protocol. It is based on the analysis and incorporation of real-time RTT measurements in the time synchronization process. The comparison of different strategies (GNTP, SNTP, and DNTP) for time synchronization has validated the proposal. The results have been obtained in terms of the mean, variance, median, and standard deviation of each protocol. It can be concluded from the results that both the GNTP and DNTP approaches outperform SNTP by providing lower values of variance and standard deviation and higher values for median accuracy. Nevertheless, a higher mean value has been observed for the GNTP and the DNTP approaches as compared to the SNTP, which is primarily due to the specific conditions of their operational environment. It has also been observed from the results that the DNTP approach shows higher variation, standard deviation, and mean accuracy values as compared to the GNTP method. However, it is only the DNTP method that can adapt to changing network conditions. Therefore, it is the right choice for precise, adaptable, and consistent synchronization in varying network conditions. On the other hand, SNTP is appropriate for networks with little variations. As far as GNTP is concerned, it achieves precise time synchronization by utilizing signals from the Global Positioning System. This method is particularly effective for applications where such high precision is essential.

The work in this article has set the stage for improving the precision and dependability of timekeeping in complex computer networks with fluctuating conditions. Further research could concentrate on optimizing the method for even greater accuracy and efficiency in a variety of network situations, building on the DNTP approach. Another interesting avenue is to explore the scalability of DNTP in larger and more complex network systems. Furthermore, investigating cutting-edge machine learning methods to anticipate and adjust to network changes may greatly improve the protocol’s performance.

## Figures and Tables

**Table 1:** Comparison of NTP approaches for time synchronization.

Approach	Variance ( μ $s^{2}$ )	Std Dev ( μ s)	Mean Accuracy ( μ s)	Median Accuracy ( μ s)
Dynamic NTP	$2.370\times 10^{8}$	$1.540\times 10^{4}$	$6.0642\times 10^{4}$	66,956
Static NTP	$7.422\times 10^{8}$	$2.724\times 10^{4}$	$8.899\times 10^{3}$	$-8.380\times 10^{3}$
GPS-based NTP	$7.663\times 10^{6}$	$2.768\times 10^{3}$	$5.142\times 10^{4}$	$1.8251\times 10^{4}$

## Data Availability

Data are contained within the article.
